# Personality as Assessed by Egogram is a Possible Independent Predictive Variable for Post-discharge Smoking Abstinence in Male Cancer Patients

**DOI:** 10.2188/jea.13.303

**Published:** 2007-11-30

**Authors:** Hideo Tanaka, Seiko Hasuo, Shigeko Matsuo, Sachiko Housou, Setsuko Numanami, Akira Oshima, Naoyuki Okamoto

**Affiliations:** 1 Department of Cancer Control and Statistics, Osaka Medical Center for Cancer and Cardiovascular Diseases; 2 Department of Nursing, Osaka Medical Center for Cancer and Cardiovascular Diseases; 3 Department of Epidemiology, Kanagawa Cancer Research Institute

**Keywords:** smoking cessation, neoplasms, personality, egogram, cohort studies

## Abstract

BACKGROUND: Little is known about the relationship between a patient’s personality and smoking behavior.

METHODS: We assessed the smoking status of 262 male smokers who had been diagnosed with cancer and admitted to a teaching hospital, using a self-administered questionnaire that was mailed to the patients 6 months after discharge. The personality of the patients was assessed with the Kyushu University Egogram at admission, and the patients were categorized into five groups according to the ego state with the highest value among the five ego states, namely “Critical Parent” dominant, “Nurturing Parent” dominant, “Adult” dominant, “Free Child” dominant and “Adapted Child” dominant. Multivariate logistic regression analyses were used to assess the influence of the type of personality on smoking behavior after hospital discharge with adjustment for considerable predictive variables.

RESULTS: The smoking cessation rate at 6 months after hospital discharge was 63% (164/262). Multivariate analyses revealed that after adjustment for age, cancer site, length of hospital stay, time elapsed since last cigarette, self-confidence to quit smoking and strength of nicotine dependence, and being an Adult dominant personality were positively (p<0.01), and being a Free Child dominant personality was negatively (p<0.05) associated with post-discharge abstinence. These findings did not change when the non-responders (n=50) of the questionnaire were included in the analysis as post-discharge smokers.

CONCLUSIONS: These findings indicate that a male cancer patient’s personality as assessed by the egogram has predictive significance for whether the patient will have a smoking habit after discharge.

Continued smoking following diagnosis of a smoking-related cancer adversely affects the general health of the cancer patient and increases the risks for treatment-related complications,^1^^,^^2^ a second primary cancer^3^^-^^5^ and mortality.^1^ Assessment of the smoking status after diagnosis of cancer and identification of predictors of whether the patient will continue smoking are important in the management of cancer patients.

We previously described the change in smoking behavior of cancer patients after diagnosis and admission, and attempted to elucidate factors associated with post-discharge abstinence.^6^ In that study, we had the impression that those patients who had succeeded in refraining from smoking after the diagnosis of cancer had some common characteristics in their personality or behavior; they tended to be rational, intelligent, temperate, and serene. We hypothesized that these characteristics of the mental state may play a role in the behavioral change of smoking after diagnosis of cancer. The current study was conducted to find the potential predictive factors on smoking cessation in cancer patients with special attention to the patients’ personality, which has not been well examined in relation to smoking behavior. The purpose of the study was to assess whether a certain type(s) of personality as characterized by the egogram is an independent predictive factor for post-discharge abstinence in cancer patients.

## METHODS

### Patient recruitment

**Figure 1.**
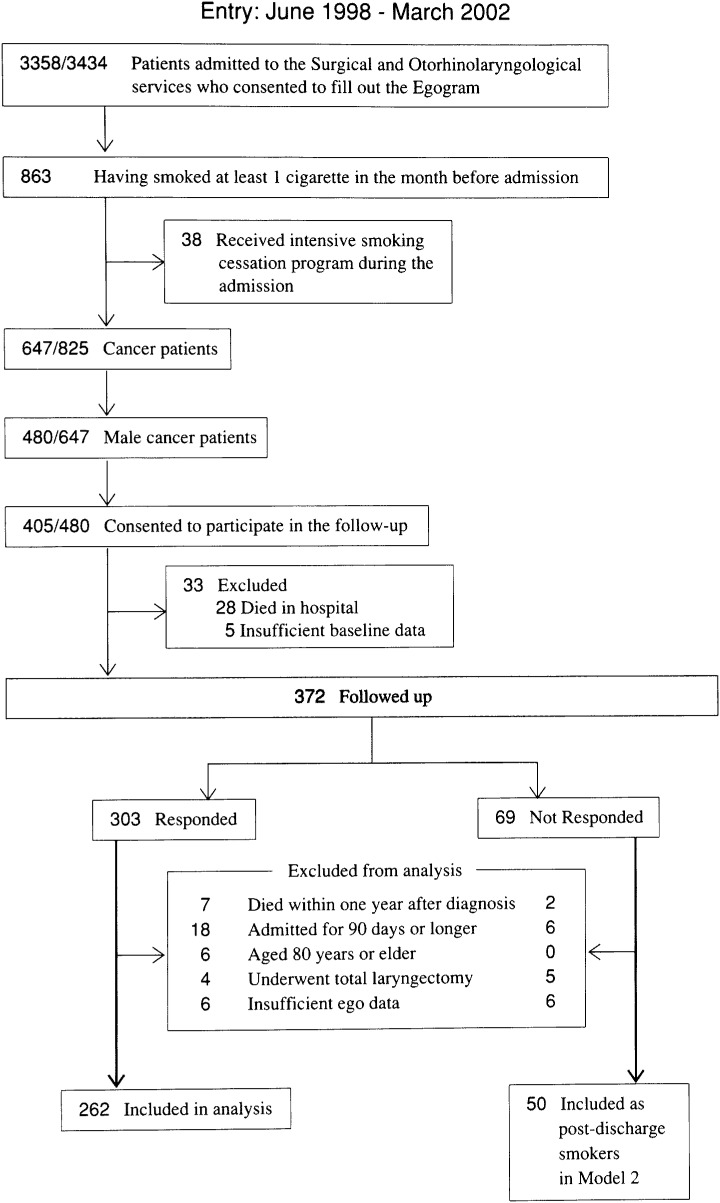
Process of selecting the study subjects

This study included patients who were diagnosed with cancer and who were admitted to the surgical and otorhinolaryngological services at Osaka Medical Center for Cancer and Cardiovascular Diseases between June 1998 and March 2002. We assessed each patient’s personality at the beginning of admission by asking the patient to fill out the Kyushu University Egogram. The purposes of asking the patient to fill out the Egogram were to use it in nursing care and to explore the relationship between a patient’s personality and the patient’s health behavior after discharge from the hospital. A total of 3,434 patients were newly admitted to the services during the study period. Among the 3,358 patients who consented to fill out the Egogram, 863 had smoked at least 1 cigarette in the month before the admission. Of these, 38 patients underwent an intensive smoking cessation program consisting of a bedside counseling session by a trained nurse. We identified 480 male cancer patients in the remaining 825 patients. They were asked to reply to a questionnaire about the smoking status that would be mailed to them after they were discharged, and they were given a leaflet to stop smoking. Four hundred five patients (84%) agreed to reply to the questionnaire, but 33 of them were excluded due to death in the hospital (n=28) or insufficient baseline information (n=5) after he gave consent. We mailed a self-administered questionnaire to the remaining 372 patients at 6 months after discharge, and 303 individuals (81%) returned a completed questionnaire. Out of the 372 patients, we excluded those who died within one year after discharge, those who were admitted for 90 days or longer, those aged 80 years or older, those who underwent total laryngectomy, and those in whom we had insufficient ego data (Figure 1). The remaining 262 patients who returned a completed questionnaire and 50 patients who did not return a questionnaire (the responders and non-responders, respectively) were included in the analysis. Female patients were excluded because of the relatively small number of those who smoked and the possible interaction of sex in the relationship between personality and smoking behavior. The cancer notification rate during the hospital admission has reached 90% at the end of the 1990s.^7^

### Assessment of personality

Egogram is used with Transactional Analysis, which is one of the methods of psychotherapy. The Transactional Analysis was developed by Eric Berne.^8^ The basic general idea in the Transactional Analysis is that our consciousness is composed of five ego states (Critical Parent: CP, Nurturing Parent: NP, Adult: A, Free Child: FC, Adapted Child: AC). Each person’s personality is thought to be expressed by the power of these five ego states. Dusay, who was a follower of Berne, developed a questionnaire to assess these five ego states^9^. The five ego states measured by this questionnaire are referred to as the egogram. The five ego states of the egogram are defined as follows. The Critical Parent manifests itself in moralizing, criticizing, judgmental attitudes, punitive actions, and deep concern for law and order. The Nurturing Parent is supportive of others, concerned, protective, and nurturing, both in language and in nonverbal behavior. The Adult is an ego state oriented toward objective, autonomous data-processing, and probability estimating. The Free Child includes the following characteristics: sensuous, affectionate, uncensored, curious, impulsive, self-indulgent and self-centered, creative and spontaneous, and exhibiting the primitive impulses of sex, aggression, grief, and desire for food. The Adapted Child behaviors include strict compliance, withdrawing, procrastinating, fear of sex, low self-concept, extremes of pleasing, and submission.^9^^,^^10^

To assess the personality of each cancer patient, we adopted the Kyushu University Egogram^11^ which was developed by Shinzato.^12^ This self-administered questionnaire form is comprised of 30 items, which are listed in the Appendix. The main reason we adopted this Egogram in our hospital was that the number of the questionnaires is relatively smaller than other egograms like the Tokyo University Egogram,^13^ and it has already been used as a self-administered questionnaire in one of the Japanese cancer center.^14^ Each of the five ego states is assessed by six items. In the questionnaire, the subjects were asked how strongly they agreed with each statement, by marking “strongly agree”, “agree”, “no opinion”, “disagree”, or “strongly disagree”, which were scored as 5, 4, 3, 2, or 1 point, respectively.^12^ The points on the items in each ego state category were added, and the total number of points in each ego state category was transformed to the deviation value according to the standardized scale that had been constructed on the basis of data from 979 healthy Japanese individuals. In the present study, the patients were categorized into five groups according to the ego state with the highest relative value among the five ego states in that individual, namely “Critical Parent dominant”, “Nurturing Parent dominant”, “Adult dominant”, “Free Child dominant” or “Adapted Child dominant”. We adopted this grouping on the basis of a former study that assessed the relationship between smoking habit and grouping using the Kyushu University Egogram among 918 ischemic heart disease patients.^15^

### Data collection and assessment of outcome

Baseline information was obtained using a self-administered questionnaire which the patient was required to fill out on admission, and included (1) demographic factors, (2) past and recent smoking habits, (3) 6 questionnaires to assess the Fagerström Test for Nicotine Dependence (FTND),^16^ (4) readiness to stop smoking, and (5) confidence in ability (self-confidence) not to smoke after discharge. The patients responded to the question, “How confident are you that you will not smoke during the one-year period after discharge?”, on a scale from 0% (not at all) to 100% (very) with 11 grades. Information on the length of hospital stay, discharge diagnoses and surgical procedures was collected by chart review. Smoking status and experience with nicotine replacement therapy after hospital discharge were assessed using a self-administered questionnaire that was mailed to the patients 6 months after discharge from the hospital. As to smoking habit, the patient was asked whether he had smoked at least 1 cigarette in the week after discharge from the hospital, and in the past week. To patients who failed to reply within 10 days, we mailed a reminder and the questionnaire. A patient with post-discharge abstinence was defined as a patient who had not smoked both during the week after discharge and during the past week without any nicotine replacement therapy. This study protocol was approved by the Ethical Committee of Osaka Medical Center for Cancer and Cardiovascular Diseases.

### Statistical analyses

The chi-square test or Wilcoxon’s signed rank test was used to compare the frequency of the baseline characteristics between the responders and non-responders. The chi-square test was used to test the distribution of the baseline characteristics of the patients in the five ego groups. The difference in the rate of smoking abstinence after discharge according to age, cancer site, length of hospital stay, time elapsed since last cigarette, the number of cigarettes per day, FTND score, self-confidence in smoking abstinence after discharge, and the egogram-based grouping were tested by the chi-square test. We used logistic regression analyses to generate odds ratios (ORs) and their 95% confidence intervals (CIs) to adjust the effect of ego pattern for potential confounding factors. First, univariate analyses were performed to identify baseline characteristics that were associated with a smoking habit after hospital discharge. Then, factors associated with cessation on the univariate analyses at a level of p<0.10 were retained and the factor of 5 egogram-based groupings was included one by one into a multiple logistic regression model. We used the 5 egogram-based groupings in the analyses as to clarify the difference of smoking behavior by the presence or absence of each dominant personality. The dependent variable was post-discharge abstinence. The models were adjusted for age, site of cancer, length of hospital stay, elapsed time since the last cigarette at the hospital admission, self-confidence for post-discharge abstinence, and the FTND score. The FTND score, known as a risk factor of smoking behavior, would be included in the independent variable even if it had not a statistically significant association in the univariate analysis. We used the data of the 262 responders in the multivariate analyses of Model 1. The 50 patients who did not respond to the follow-up mailing but who satisfied the eligible criteria (the non-responders) were considered as post-discharge smokers, and were included in the multivariate analyses (Model 2). A p value less than 0.05 was considered to indicate statistical significance.

## RESULTS

Table 1 summarizes the baseline characteristics of the responders and non-responders. Significantly higher proportions of the responders had quit smoking between 4 to 30 days before the admission, had high self-confidence for post-discharge abstinence, and were aged 65 years or older (p=0.001, p=0.009, p=0.001). There were no significant differences between the responders and non-responders with regard to the cancer site, length of hospital stay, the number of cigarettes per day, FTND score, and the grouping of the egogram. Regarding the results of the Egogram, 81 patients (31%) among the 262 responders were Adult dominant, 73 (28%) were Critical Parent dominant, 46 (17%) were Adapted Child dominant, 41 (16%) were Nurturing Parent dominant, and 21 (8%) were Free Child dominant (Table 2). There were no significant differences in age, cancer site, length of hospital stay, the number of cigarettes per day, nor self-confidence about post-discharge abstinence among the 5 egogram groups. Marginally significant differences were observed in the distribution of the time elapsed since the last cigarette (p=0.07) and the FTND score (p=0.067) among the 5 groups. Only 34% of the Nurturing Parent dominant patients had quit smoking more than 3 days before the admission, whereas 60% of the Adult dominant patients had. Those patients who were Adapted Child dominant had a significantly higher dependence on nicotine than patients who were Adult dominant or Free Child dominant (p<0.01, p<0.01 with Wilcoxon’s signed rank test).

**Table 1.** Baseline characteristics of the responders and non-responders.

	Responders	Non-responders	p value
	n= 262	n= 50	
Age (year)			0.001 ^#^
-54	69 (26)	14 (28)	
55-64	92 (35)	31 (62)	
65-	101 (39)	5 (10)	
Cancer site			0.109^#^
Esophagus	26 (10)	0 (0)	
Lung	44 (17)	5 (10)	
Head and Neck	89 (34)	20 (40)	
Stomach	54 (21)	12 (24)	
Colon and rectum	19 (7)	7 (14)	
Liver	18 (7)	5 (10)	
Others	12 (5)	1 (2)	
Hospital stay (day)			0.859^# #^
-21	89 (34)	20 (40)	
22-30	66 (25)	8 (16)	
31-	107 (41)	22 (44)	

Time elapsed since last cigarette (day)			0.001 ^#^
4-30	136 (52)	12 (24)	
≤3	126 (48)	38 (76)	

Number of cigarettes per day			0.476^#^
-20	159 (61)	29 (58)	
21-	92 (35)	21 (42)	
Not available	11 (4)	0 (0)	
FTND score			0.212^# #^
0-3	84 (32)	11 (22)	
4-5	74 (28)	18 (36)	
6-10	93 (35)	21 (42)	
Not available	11 (4)	0 (0)	
Self-confidence about post-discharge abstinence (%)			0.009^# #^
0-30	51 (19)	14 (28)	
40-60	62 (24)	18 (36)	
70-100	135 (52)	15 (30)	
Not available	14 (5)	3 (6)	

Grouping of the egogram			0.380^#^
CP dominant	73 (28)	15 (30)	
NP dominant	41 (16)	5 (10)	
A dominant	81 (31)	17 (34)	
FC dominant	21 (8)	1 (2)	
AC dominant	46 (18)	12 (24)	

^#^Chi-square test ^##^Wilcoxon’s signed rank test

CP:Critical Parent. NP:Nurturing Parent. A:Adult. FC:Free Child. AC:Adapted Child.

FTND:Fagerström Test for Nicotine Dependence

Percentages in parentheses

**Table 2.** Comparison of baseline characteristics related to smoking behavior among male cancer patients according to the grouping of the egogram.

		Grouping of the egogram					
	
Characteristic	Total	CP dominant	NP dominant	A dominant	FC dominant	AC dominant	p Value ^†^
	n= 262	n= 73	n= 41	n= 81	n= 21	n= 46	
Age (year)							0.552
-54	69 (26)	22 (30)	11 (27)	18 (22)	8 (38)	10 (22)	
55-64	92 (35)	26 (36)	16 (39)	24 (30)	7 (33)	19 (41)	
65-	101 (39)	25 (34)	14 (34)	39 (48)	6 (29)	17 (37)	
Cancer site							0.575
Esophagus	26 (10)	5 (7)	4 (10)	7 (9)	4 (19)	6 (13)	
Lung	44 (17)	9 (12)	7 (17)	20 (25)	1 (5)	7 (15)	
Head and Neck	89 (34)	28 (38)	12 (29)	21 (26)	11 (52)	17 (37)	
Stomach	54 (21)	20 (27)	8 (20)	14 (17)	3 (14)	9 (20)	
Colon and rectum	19 (7)	6 (8)	3 (7)	7 (9)	0 (0)	3 (7)	
Liver	18 (7)	3 (4)	5 (12)	7 (9)	1 (5)	2 (4)	
Others	12 (5)	2 (3)	2 (5)	5 (6)	1 (5)	2 (4)	
Hospital stay (day)							0.240
-21	89 (34)	27 (37)	12 (29)	32 (40)	9 (43)	9 (20)	
22-30	66 (25)	18 (25)	12 (29)	17 (21)	2 (10)	17 (37)	
31-	107 (41)	28 (38)	17 (41)	32 (40)	10 (48)	20 (43)	

Time elapsed since last cigarette (day)							0.070
4-30	136 (52)	35 (48)	14 (34)	49 (60)	12 (57)	26 (57)	
≤3	126 (48)	38 (52)	27 (66)	32 (40)	9 (43)	20 (43)	
Number of cigarettes per day							0.109
-20	159 (61)	42 (58)	27 (66)	57 (70)	10 (48)	23 (50)	
21-	92 (35)	29 (40)	12 (29)	21 (26)	9 (43)	21 (46)	
Not available	11 (4)	2 (3)	2 (5)	3 (4)	2 (10)	2 (4)	
FTND score							0.067
0-3	84 (32)	19 (26)	13 (32)	35 (43)	6 (29)	11 (24)	
4-5	74 (28)	22 (30)	10 (24)	26 (32)	6 (29)	10 (22)	
6-10	93 (35)	30 (41)	16 (39)	17 (21)	7 (33)	23 (50)	
Not available	11 (4)	2 (3)	2 (5)	3 (4)	2 (10)	2 (4)	
Self-confidence about post- discharge abstinence (%)							0.722
0-30	51 (19)	14 (19)	10 (24)	14 (17)	3 (14)	10 (22)	
40-60	62 (24)	22 (30)	10 (24)	16 (20)	7 (33)	7 (15)	
70-100	135 (52)	35 (48)	20 (49)	45 (56)	11 (52)	24 (52)	
Not available	14 (5)	2 (3)	1 (2)	6 (7)	0 (0)	5 (11)	

† : Chi-square test

CP:Critical Parent. NP:Nurturing Parent. A:Adult. FC:Free Child. AC:Adapted Child.

FTND:Fagerströdm Test for Nicotine Dependence

Percentages in parentheses

Among the responders, the post-discharge abstinence rate was 63% (164 /262) (Table 3). Patients who received nicotine replacement therapy (10 received gums and 3 received patches) after hospital discharge were considered as smokers in this study. Longer hospital stay, time elapsed since last cigarette of 4 to 30 days, having self-confidence in post-discharge abstinence, and grouping of the egogram were significantly associated with smoking cessation after hospital discharge. Patients who were Adult dominant had the highest smoking cessation rate (78%) among the 5 egogram groups, and patients who were Free Child dominant had the lowest smoking cessation rate (43%). Neither the number of cigarettes per day nor the FTND score significantly affected smoking cessation. When the non-responders were included as post-discharge smokers, the smoking cessation rate decreased to 53% (164/312). Factors that were significantly associated with smoking cessation were old age, time elapsed since the last cigarette of 4 to 30 days, and self-confidence in post-discharge abstinence. The site of a cancer related to smoking (esophageal, lung, head and neck), longer hospital stay, and grouping of the egogram were associated with smoking cessation at a marginally significant level.

**Table 3.** Abstinence from cigarettes 6 months after hospital discharge in male cancer patients according to the baseline characteristics and the grouping of the egogram.

	Model 1			Model 2		
	
	Smoking cessation		P	Smoking cessation		P
Total	164 / 262	(63)		164 / 312	(53)	
Age (year)			0.073			0.001
-54	43 / 69	(62)		43 / 83	(52)	
55-64	50 / 92	(54)		50 / 123	(41)	
65-	71 / 101	(70)		71 / 106	(67)	
Cancer site			0.153			0.056
Smoking-related cancer^†^	105 / 159	(66)		105 / 184	(57)	
Other cancer	59 / 103	(57)		59 / 128	(46)	
Hospital stay (day)			0.041			0.060
-21	50 / 89	(56)		50 / 109	(46)	
22-30	39 / 66	(59)		39 / 74	(53)	
31-	75 / 107	(70)		75 / 129	(58)	

Time elapsed since last cigarette (day)			0.001			0.001
4-30	113 / 136	(83)		113 / 148	(76)	
≤3	51 / 126	(40)		51 / 164	(31)	
Number of cigarettes per day			0.507			0.801
-20	97 / 159	(61)		97 / 188	(52)	
21-	60 / 92	(65)		60 / 113	(53)	
Not available	7 / 11	(64)		7 / 11	(64)	
FTND score			0.301			0.131
0-3	55 / 84	(65)		55 / 95	(58)	
4-5	48 / 74	(65)		48 / 92	(52)	
6-10	54 / 93	(58)		54 / 114	(47)	
Not available	7 / 11	(64)		7 / 11	(64)	
Self-confidence about post- discharge abstinence (%)			0.001			0.001
0-30	16 / 51	(31)		16 / 65	(25)	
40-60	29 / 62	(47)		29 / 80	(36)	
70-100	108 / 135	(80)		108 / 150	(72)	
Not available	11 / 14	(79)		11 / 17	(65)	

Grouping of the egogram			0.009			0.080
CP dominant	43 / 73	(59)		43 / 88	(49)	
NP dominant	22 / 41	(54)		22 / 46	(48)	
A dominant	63 / 81	(78)		63 / 98	(64)	
FC dominant	9 / 21	(43)		9 / 22	(41)	
AC dominant	27 / 46	(59)		27 / 58	(47)	

Model 1: The responders who returned a completed questionnaire on post-discharge abstinence were entered in the analysis.

Model 2: Non-responders (n=50) who satisfied the eligible criteria, were included in the analysis as having failed to quit smoking after discharge.

† : Cancer of esophagus, lung, or head and neck

FTND:Fagerström Test for Nicotine Dependence

CP:Critical Parent. NP:Nurturing Parent. A:Adult. FC:Free Child. AC:Adapted Child.

Percentages in parentheses

Univariate analyses of the baseline characteristics using a logistic regression model revealed that age of 55-64 years (55-64 years/others, p<0.02), longer hospital stay (31 days or longer/30 days or shorter, p<0.07), site of smoking-related cancer (smoking-related cancer/others, p<0.05), time elapsed since last cigarette of 4 to 30 days (4-30 days/less than 4 days, p<0.001), and high self-confidence (70% or stronger/60% or weaker, p<0.001) were significant or marginally significant predictors of post-discharge abstinence. Finally, we performed multivariate analyses using 7 independent variables including the six factors and the factor of 5 egogram-based groupings, adding each variable one by one (Table 4). In Model 1, patients with an Adult dominant personality had a significantly higher post-discharge smoking cessation rate than those in the other ego groups (OR=2.78, 95% CI=1.31-5.91). Conversely, patients with a Free Child dominant personality had a significantly lower post-discharge smoking cessation rate than the other ego groups (OR=0.33, 95% CI=0.11-0.95). In Model 2, the OR for Adult dominant was significantly higher than unity (p=0.03), and the OR for Free Child dominant was lower than unity at a marginally significant level (p=0.09).

**Table 4.** Type of personality as assessed by the egogram and post-discharge smoking abstinence among male cancer patients: multivariate logistic regression analysis.

	Model 1 (n=262)		Model 2 (n=312)	
	
	odds ratio (95% CI)	p	odds ratio (95% CI)	p
CP dominant	0.90 (0.46-1.76)	0.75	0.99 (0.54-1.82)	0.97

NP dominant	0.90 (0.39-2.06)	0.80	1.16 (0.53-2.54)	0.70

A dominant	2.78 (1.31-5.91)	<0.01	2.27 (1.10-4.79)	0.03

FC dominant	0.33 (0.11-0.95)	<0.05	0.43 (0.19-1.17)	0.09

AC dominant	0.63 (0.27-1.50)	0.29	0.65 (0.31-1.39)	0.26

Seven independent variables including the six baseline characteristics (Age:55-64 years/others, Cancer site: esophagus, lung, head and neck/others, hospital stay:31 days or longer/others, time elapsed since last cigarette: 4-30 days/≤3 days, self-confidence about post-discharge abstinence:70% or stronger/60% or weaker, FTND score: 0-3/4-5/6-10) were used in the analyses.

Model 1: The responders who returned a completed questionnaire on post-discharge abstinence were entered in the analysis.

Model 2: Non-responders (n=50) who satisfied the eligible criteria, were included in the analysis as having failed to quit smoking after discharge.

CI:Confidence Intervals

CP:Critical Parent. NP:Nurturing Parent. A:Adult. FC:Free Child. AC:Adapted Child.

## DISCUSSION

Several studies have assessed the relationship between personality and smoking-associated factors.^17^^-^^19^ Sensation seeking and/or impulsivity was reported to be a possible risk factor for nicotine dependence in men^18^ and women.^17^ However, little is known as to whether a certain type of personality influences one’s smoking behavior. If some types of personality affect the change in smoking behavior, this knowledge may be useful in the management of smoking cessation. To our knowledge, this is the first report to describe the relationship between personality and the behavior of smoking cessation in a cohort study.

Analyses of patients who returned a completed questionnaire on their post-discharge smoking habit indicated that being Adult dominant or being Free Child dominant was each a significant independent predictor of smoking cessation behavior in male cancer patients after hospital discharge. The response rate to the mailed questionnaire including the second questionnaire with a reminder was 75%. The smoking cessation rate among the non-responders was probably lower than that among the responders because the non-responders had lower smoking cessation predictors of low self-confidence and shorter time elapsed since last cigarette than the responders. Therefore, we added the 50 non-responders who satisfied the eligible criteria into the analysis and found nearly consistent results as to the significance of Adult dominant personality (p=0.03) and Free Child dominant personality (p=0.09). Since a person with a personality characterized by “Adult” performs realistic problem solving, it may be considered that Adult dominant individuals are more likely to change their behavior once they have a serious illness such as cancer than those who have the other dominant ego-types. Free Child dominant patients are often outspoken in their remarks, behave just as they like, and are not obedient. Further studies are needed to identify the possible relationship between the characteristics of Free Child and other aspects of personality, i.e., impulsivity and disinhibition,^17^^,^^18^ which may be potential confounding factors on negative health behaviors.

Our study involves some methodological limitations. First, according to a previous study,^14^ the performance status of cancer patients may influence the ego status. Okamoto et al.^14^ explored the relationship between the Functional Living Index for Cancer and the five ego states among 62 patients with cancer who filled out the questionnaire on the ego states at the time of admission. They found a positive correlation between somatic discomfort as determined by the Functional Living Index for Cancer and the Adapted Child score. It may be realistic to consider that cancer patients who have severe somatic discomfort at the time of hospital admission are more likely to abstain from smoking after discharge than those who do not. Therefore, a somatic influence on the characteristics of Adapted Child might underestimate the present finding of the advantage of Adult dominant cancer patients in quitting smoking, if it exists. On the contrary, the possible confounder of somatic discomfort in the relationship between the egogram and smoking behavior would overestimate the present finding of a lower smoking cessation rate among Free Child dominant patients. We therefore performed the multivariate logistic regression analysis excluding the Adapted Child dominant patients, in the calculation of the OR for being Free Child dominant and found a similar result (OR=0.23, p<0.01 in Model 1; OR=0.36, p=0.05 in Model 2). We did not report the change in the ego state of patients in a follow-up questionnaire that the patients filled out. If reproducibility of the individuals’ egogram during the follow-up period is not obtained, it would cause misclassification of ego state, although the egogram was reported to be stable across a time interval.^20^

Second, in patients who replied that they had quit smoking in the questionnaire, we did not confirm that the patients had actually quit smoking by examining biomarkers. However, since the investigators did not have an opportunity to see the study subjects after discharge, there was no incentive for the smokers to answer falsely. If some ego states are associated with the behavior of sending a false reply, such misclassification would introduce a bias in the strength of the association between some ego states and post-discharge smoking abstinence among the study subjects.

Third, other potential confounding factors that were not considered in this study were clinical stage at diagnosis, complications, habitual drinking, and education level, which should be considered in future studies.

The results of our study indicate that the personality of the patient, as characterized by the egogram, is a predictive variable in the behavior of smoking cessation in cancer patients. However, since this study was performed in Japanese male cancer patients, further studies that consider various clinical features and/or patients of other races are needed. Interventional studies are also needed to clarify the causal relationship between the type of personality and smoking behavior. Since transactional analysis is a theory regarding personality and psychotherapy,^10^ psychiatric interventions in combination with ego-specific materials for smoking cessation may be effective. We also believe that further studies on the relationship between the type of personality and smoking behavior may lead to the development of tailored smoking cessation programs.

## Appendix: Items in the Kyushu University Egogram ^†^

**Table:**

Items concerning Critical Parent (CP). 6 items
1. I persist in my opinion.
6. I don’t change my position on matters easily.
11. I am in the habit of taking on a leadership role.
16. When I talk, I use numbers and data.
21. I can say what I think without feeling constrained by people around me.
26. I don’t often take action positively of my own accord. ^† †^

Items concerning Nurturing Parent (NP). 6 items
2. When I see someone in trouble, I want to help him/her.
7. I like to give a helping hand to other people.
12. Other people often say to me, “I feel relieved when you’re with me”.
17. I can empathize with other people and feel their happiness and sadness.
22. I feel happy working voluntarily for other people.
27. I always judge surrounding people’s feelings sensitively when taking action.

Items concerning Adult (A). 6 items
3. I look at things objectively, according to the facts.
8. I judge all things calmly after gathering information.
13. I’m rational rather than emotional.
18. When I shop, I plan ahead and hardly ever make impulse purchases.
23. Other people often speak to me.
28. I don’t show disagreeable feelings in my expression or words, but put on a smiling face.

Items concerning Free Child (Free Child). 6 items
4. I have nothing against an atmosphere of fun and can throw myself into it.
9. I often go to movies, shows and so on.
14. I can forget myself in playing with children and animals.
19. I don’t mind spending money on enjoyable activities for myself.
24. I can do unconventional things without feeling bad about it.

Items concerning Adapted Child (AC). 6 items
5. I always make great effort to make other people think well of me.
10. I don’t have confidence in my own thoughts or actions.
15. When things don’t go well, I tend to blame other people.
20. I can hold grudges against people for a long time.
25. I get trapped in a sense of regret that doesn’t go away.
30. I am resigned to not being able to be as happy as other people are.

The number placed at the beginning of each item represents the order in which the items are listed in the Kyushu University Egogram.

† : The items in the Kyushu University Egogram were translated from Japanese to English by a native English speaker who has a background in psychology.

† † : The score was converted by subtracting the score from 6.
